# Preoperative education in patients undergoing foot and ankle surgery: a scoping review

**DOI:** 10.1186/s13643-023-02375-2

**Published:** 2023-11-13

**Authors:** Laura Vergara-Merino, María Jesús Lira, Camila Micaela Escobar Liquitay, Nicolás González-Kusjanovic, Sergio Morales

**Affiliations:** 1 Orthopedic Surgery Department, Pontificia Universidad Católica de Chile, Diagonal Paraguay 362, 8330077 Santiago, Chile; 2 Research Department, Associate Cochrane Centre, Instituto Universitario Escuela de Medicina del Hospital Italiano de Buenos Aires, Buenos Aires, Argentina

**Keywords:** Patient education as topic, Orthopedics, Foot and ankle surgery, Review

## Abstract

**Background:**

International guidelines promote preoperative education for patients undergoing orthopedic surgery. However, the evidence sustaining these recommendations comes mainly from studies for hip and knee replacement surgery. Little is known about patients undergoing foot and ankle surgery. We aimed to map and characterize all the available evidence on preoperative education for patients undergoing foot and ankle surgery.

**Methods:**

This study complies with the PRISMA-ScR guidelines. We searched eight databases, including MEDLINE, Embase, and CENTRAL. We performed cross-citations and revised the references of included studies. We included studies addressing preoperative education in patients undergoing foot and ankle surgery. We did not exclude studies because of the way of delivering education, the agent that provided it, or the content of the preoperative education addressed in the study. Two independent authors screened the articles and extracted the data. The aggregated data are presented in descriptive tables.

**Results:**

Of 1596 retrieved records, only 15 fulfilled the inclusion criteria. Four addressed preoperative education on patients undergoing foot and ankle surgery and the remaining 11 addressed a broader population, including patients undergoing foot and ankle surgery but did not provide separate data of them. Two studies reported that preoperative education decreases the length of stay of these patients, another reported that education increased the knowledge of the participants, and the other leaflets were well received by patients.

**Conclusion:**

This scoping review demonstrates that evidence on preoperative education in foot and ankle surgery is scarce. The available evidence supports the implementation of preoperative education in patients undergoing foot and ankle surgery for now. The best method of education and the real impact of this education remain to be determined.

**Supplementary Information:**

The online version contains supplementary material available at 10.1186/s13643-023-02375-2.

## Background

Preoperative education refers to any educational process that healthcare professionals deliver to patients before surgery to improve their knowledge of the procedure, health behaviors, and clinical outcomes [1, 2]. It may have several benefits, such as reducing patients’ anxiety, lowering postoperative pain, improving patients’ satisfaction, and improving other outcomes depending on the performed surgery [2–7]. There are many ways to deliver education to patients: perform face-to-face teaching, yield written or pictorial information, establish surgery schools, and produce digital data (e.g., DVDs or online videos), among others [2]. The education content may vary between health centers and by the patient’s illness.

The Enhanced Recovery After Surgery (ERAS) Society suggests that education should be carried out with a multidisciplinary approach in orthopedics surgery: with physiotherapists, occupational therapists, and nurses [2]. It should consider “joint schools” and be undertaken by small groups focusing on patient expectations and postoperative recovery [2].

Mostly, evidence from preoperative education for total hip and knee arthroplasty studies supports these guidelines [8–13]. Multiple studies addressing preoperative education for total hip and knee have been published and widely diffused later [14–17]. However, evidence specifically regarding preoperative education for patients undergoing foot and ankle surgery is less widespread and has not been summarized yet.

There is a vast spectrum of different foot and ankle orthopedic surgeries, with a high rate of elective procedures [18]. Patients undergoing elective foot and ankle procedures may be exposed to preoperative education, but there is no evidence-based recommendation to guide education in this group of patients.

This scoping review aims to identify and describe all the available evidence addressing preoperative education in patients undergoing foot and ankle surgery.

## Methods

### Study design

This manuscript is a scoping review addressing the studies about preoperative education on patients undergoing foot and ankle surgery. It complies with the PRISMA-ScR extension for reporting scoping reviews [19]. A protocol for this study was previously published in Open Science Framework [20].

### Eligibility criteria

We included all published and ongoing available studies addressing preoperative education on patients undergoing foot and ankle surgery. We also included all available studies addressing preoperative education on patients undergoing any orthopedic surgery, including those patients undergoing foot and ankle surgery, even if they do not present separate data from this population.

We considered preoperative education any intervention aimed to improve patients’ knowledge about their surgery or patient outcomes. Therefore, we excluded studies addressing how to deliver proper informed consent to the patient. We did not exclude studies because of the way of delivering education, the agent that provided it, or the content of the preoperative education addressed in the study.

We included studies written in any language; those in a language different than English, Spanish, or French were translated with Google translator.

We included every study addressing our question with any original methodological design (primary or secondary), excluding narrative reviews, opinion articles, and letters to the editor.

### Data sources and search strategies

We conducted electronic searches in databases to identify studies. Studies were not excluded based on their language of publication or publication status.

The searches were conducted in the databases below:

- MEDLINE (Ovid) (1946 to November 2021)
- Embase (Elsevier.com) (1974 to November 2021)
- Latin American and Caribbean Literature in Health Sciences (LILACS), from 1982 to November 2021)
- Education Resources Information Center (ERIC; EBSCO) (from 1966 to November 2021)
- ISI Web of Science (Clarivate, WoS) (from inception to November 2021)
- Cochrane Central Register of Controlled Trials (CENTRAL) (from inception to November 2021);

To address grey literature, we performed a cross-citation search in Google Scholar and checked the references list for each included study. We also searched for unpublished and ongoing studies in the following:

- ClinicalTrials.gov trials registry at the USA National Institutes of Health (ClinicalTrials.gov)
- World Health Organization International Clinical Trials Registry Platform (ICTRP) (trialsearch.who.int; from inception to November 2021)

Furthermore, Embase database retrieves abstracts from medical congress, which had widen our gray literature search.

All the strategies were peer reviewed by another senior information specialist prior to execution using the PRESS checklist [21]. For detailed search strategies, see Additional file 1.

We managed search results and removed duplicates in EndNote X9 (Clarivate).

### Selection of studies

Two independent reviewers screened the titles and abstracts of studies retrieved from the search with the aid of Rayyan [22]. Two independent reviewers then selected the studies by reading the full text of potentially eligible studies. If there was a discrepancy during any selection process step, a third reviewer decided whether to include or exclude the study. We registered the reasons for exclusion throughout the whole process. We present the selection process in a PRISMA flow diagram [23].

### Data extraction and presentation

Two authors independently extracted all the relevant data from included studies in a previously piloted chart. In case of disagreement, the two reviewers revised the study together and amended the data.

We extracted the following general data from all the included studies: authors, year of publication, type of publication, journal, language, country, number of participants, inclusion and exclusion criteria, and measured outcomes in each study when possible.

For selecting the data about preoperative education to be extracted, we followed the template for intervention description and replication (TIDieR) checklist and guide [24]. However, we did not extract all the items because of the specificities and scope of our study. This way, the items we extracted were as follows: the diagnosis of included participants, which surgery the patients underwent, the surgeon profession (e.g., orthopedic surgeon, podiatrist, general surgeon), the method of education delivered, used materials, who delivered the education, when it was delivered (e.g., a week before surgery, a month before surgery), where it was delivered, and the number of sessions received by the participants. Finally, we extracted the main conclusions of each included study. All the data are presented in descriptive tables.

We did not combine any results of included studies, and we did not assess the risk of bias of each included study, nor the certainty of the evidence, because this was out of the scope of our review.

## Results

**Fig. 1:**
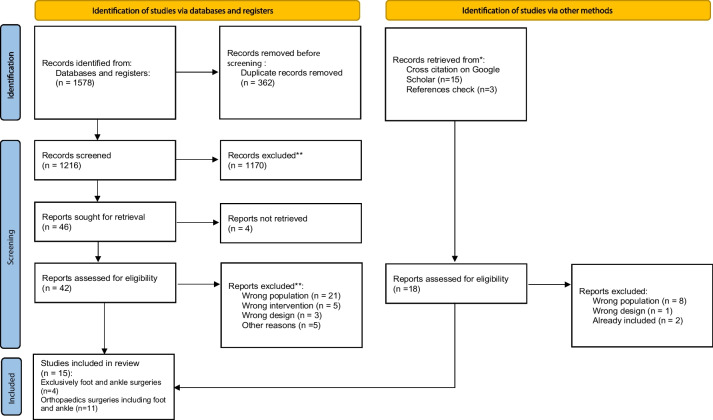
PRISMA flow diagram

Our electronic searches retrieved 1578 records. We selected 42 full-text reports after eliminating duplicates and title and abstract screening. We included eight studies after reading those 42 full text. Exclusion details are shown in Additional file 2. Also, we retrieved 18 potentially relevant studies by cross-citation or references checking, and seven of them fulfilled the inclusion criteria after full-text revision, as detailed in Additional file 3. This way, 15 studies were included in our review (Fig. 1) [25–39].

**Fig. 2:**
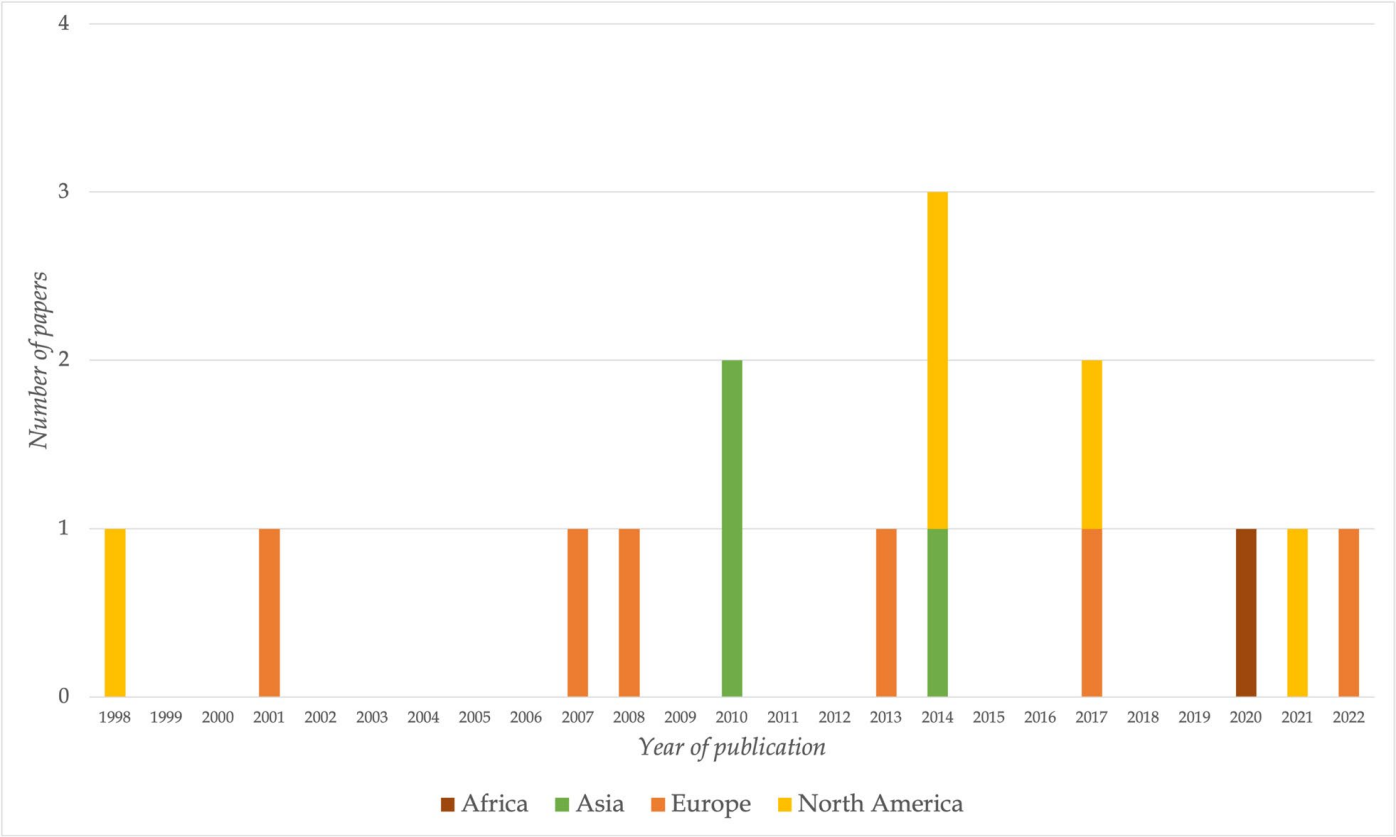
Publication date of studies by continent

The general demographics of the included studies are shown in Table 1. All but one studies were published in English [25–31, 33–39], and most of them assessed a broader population than only patients undergoing foot and ankle surgery [25–31, 33, 37–39]. Six of 15 studies were carried out in Europe [32, 34–36], five [26–29, 31] in the USA, three [37–39] in Asia, and one [30] in Africa. Figure 2 shows the date of publication of studies separated by continent. Below, we present separately the studies that exclusively address patients undergoing foot and ankle surgery (Table 2) and those addressing a broader population (Table 3).

**Table 1:** Study demographics

Characteristics	Number (%)
**Publication language**	
**English**	14 (93.3)
**German**	1 (6.7)
**Country**	
**UK**	4 (26.7)
**Germany**	1 (6.7)
**Finland**	1 (6.7)
**USA**	5 (33.3)
**Hong Kong**	3 (20.0)
**Uganda**	1 (6.7)
**Study design**	
**RCT**	3 (20)
**Quasi-experimental**	5 (33.3)
**Before and after study**	1 (6.7)
**Cross-sectional**	3 (20)
**Other observational**	3 (20)
**Addressed population**	
**Undergoing foot and ankle surgery**	4 (26.7)
**Broader population**	11 (73.3)

*Abbreviations*: *UK* United Kingdom, *USA* United States of America, *RCT* randomized controlled trial

**Table 2:** Main characteristics, evaluated interventions, and conclusions of studies exclusively addressing foot and ankle surgery

	**Speirs (2008) **[35]	**Selvan (2013) **[34]	**Schafer (2017) **[32]	**Thomas (2022) **[36]
**Journal**	The foot	Foot and ankle surgery	*International Journal of Health professions*	The foot
**Study design**	Observational	Observational (cross-sectional)	Controlled before and after	Observational
**Language**	English	English	German	English
**Country**	UK	UK	Germany	UK
**Number of participants**	161	NR	56	130 (62 attended foot school)
**Inclusion criteria**	Patients over 18 years undergoing elective foot and/or ankle surgery under local or general anesthesia, at the West Middlesex University Hospital	All foot and ankle elective patients attending for their operative intervention at Aintree University Hospital from Jan to March of 2007–2011	Patients of any age undergoing elective foot and ankle surgeries	Patients undergoing elective foot and ankle procedures from Jan 2019 to Jan 2020
**Exclusion criteria**	Revision surgery cases, pediatric cases, and patients who had insufficient understanding of English	NR	Revision surgeries and urgent surgeries	Patients who stayed overnight at the hospital due to anesthetic complications such as low blood pressure and uncontrolled pain. Insufficient data of the patient
**Diagnosis of participants**	Hallux limitus, hallux valgus, lesser toes pathology, lesser metatarsals pathology	NR	NR	NR
**Indicated surgery**	“Implant,” cheilectomy, sesamoidectomy, Lapidus procedure, osteotomies, arthroplasty, arthrodesis, Weil osteotomy, neurectomy, hemiphalangectomy, syndactylization	Only indicates if there was a forefoot, midfoot, hindfoot, ankle, fore and midfoot, or hindfoot and ankle surgery	NR	Scarf-Akin, Morton’s neuroma, Achilles-tendon lengthening, Lapidus, first MTPJ fusion, multiple toes surgery, lesser toes, calcaneal surgery
**Description of preoperative education**	Preoperative information sheets	An educative session dictated by a nurse and a physiotherapist	Training course of 2 and a half hours, with a multidisciplinary team. Introduction (10 min); education (90 min) on foot surgery, anesthesia, nursing, social services, and physical therapy care at the hospital in the treatment phase, postoperative follow-up and aid provision. “Kiosk” (60 min) practical walking exercises, physiotherapy, and social service	Group session with a physiotherapist, lead discharge nurse and providing educational material (booklets specific to their surgical procedure)
**Timing of education and number of sessions**	At the moment of informed consent	Three weeks before surgery. One session	“Before surgery.” Not detailed. One session	Within 2 weeks of surgery. One session per week
**Outcomes**	Rating of the procedure information sheet, readability of sheets	Length of stay (hours)	Increase in knowledge, satisfaction, and preoperative anxiety	Length of stay (hours)
**Main conclusions**	PILs used for foot surgery were well received by patients. The audit highlighted the areas “recovery, level of pain, and work return.” There were differences between men and women and between patients aged more and less than 60 years old	The intervention group had reduced inpatient stays, increase in day-case surgery rates with significant cost savings	The training increased the knowledge of the participants and the personal gain. The patients reported a high level of satisfaction with the process. The efficacy and profitability have to be proved in a controlled study yet to recommend the implementation	Preoperative education results in significantly shorter postoperative hospital stay, thereby saving hospital costs per procedure. Patients should, therefore, be encouraged to attend foot school before their surgical procedure

*Abbreviations*: *MTPJ* metatarsophalangeal joint, *NR* not reported, *PIL* patient information leaflet, *UK* United Kingdom

**Table 3:** Main characteristics of included studies without separate data from foot and ankle surgery exclusively

	**Journal**	**Study design**	**Language (country)**	**Number of participants**	**Included population**	**Description of preoperative education**
Heikkinen (2007) [25]	*Journal of Advanced Nursing Research*	Cross-sectional study	English (Finland)	120	Ambulatory arthroscopy surgery patients; over 18 years, Finnish speaking, no cognitive disabilities, capable of completing the questionnaire and giving informed consent	Individual face-to-face education session with a nurse. Not detailed
Holman (2014) [26]	*Journal of Orthopaedic Trauma*	Quasi-experimental	English (USA)	613	All Utah residents admitted to the orthopedic trauma service with isolated operative musculoskeletal injuries	A standardized discussion regarding opiates prescription and its limits
Ilyas (2021) [27]	Orthopedics	RCT	English (USA)	237	Patients undergoing shoulder, elbow, wrist, knee, or foot and ankle surgery	A brief multimedia presentation regarding opioids information using a handheld tablet
Laude (2017) [28]	Orthopedic nursing	Observational study. Tool implementation	English (USA)	NR	NR	Four electronic modules in a patient education system. The surgical-based education modules consisted in the following: “preparing for surgery, the surgery itself, recovering from surgery, and safety module”
Morris (2014) [29]	*Journal of Orthopaedic Trauma*	RCT	English (USA)	212 randomized, 76 analyzed	Patients 18 years or older, English speaking, with an isolated orthopedic injury requiring admission to the orthopedic trauma service and orthopedic surgery during the same admission	Delivering a biosketch card with a picture of the attending orthopedic surgeon with a brief synopsis of his educational background, specialty, surgical interests, research interests, and other interests including hobbies
Scott (2001) [33]	*Journal of Integrated Care Pathways*	Cross-sectional study	English (UK)	100	Elective orthopedic day-case patients who attended the day surgery unit over a 2-week period	Written information. Not detailed
Wong (2010a) [38]	*Journal of Advanced Nursing*	Quasi-experimental	English (Hong Kong)	125	Chinese adult, able to communicate in Cantonese, ambulatory before injury, medically diagnosed as having musculoskeletal trauma of a single limb and undergoing orthopedic surgery	A 30-min educational intervention consisting in 5 min to build up rapport with the participant, 10 min to enhance patients’ knowledge on pain and pain management, 10 min to reduce anxiety and regain self-efficacy, and 5 min to reinforcement of importance of self-efficacy
Wong (2014) [37]	Contemporary nurse	Quasi-experimental	English (Hong Kong)	152	Patients 18 years or above, ambulatory before the injury, medically diagnosed as having a single limb fracture, and undergoing orthopedic internal fixation	A 30-min educational intervention consisting in 5 min to build up rapport with the participant, 10 min to enhance patients’ knowledge on pain and pain management, 10 min to reduce anxiety and regain self-efficacy, and 5 min to reinforcement of importance of self-efficacy
Pellino (1998) [31]	Orthopedic nursing	Quasi-experimental	English (USA)	74	18 years of age and older, scheduled for an elective orthopedic surgical procedure (out an inpatients), clear sensorium, ability to read and write English	A teaching session within the learning center’s empowerment model
Wong (2010b) [39]	*Journal of Clinical Nursing*	Quasi-experimental	English (Hong Kong)	125	Patients from six orthopedic wards in two regional hospitals with fractured limb by admission roster requiring surgery	A 30-min cognitive behavioral educational intervention consisting in 5 min to build up rapport with the participant, 10 min to enhance patients’ knowledge on pain and pain management, 10 min to reduce anxiety and regain self-efficacy, and 5 min to reinforcement of importance of self-efficacy
Othin (2020) [30]	Preprint (Research Square)	RCT	English (Uganda)	398	Patients 18 years and above, scheduled for elective orthopedic surgery, which consented to participate in the study	Specific preoperative information about pain was given verbally following an order on the leaflet

*Abbreviations*: *NR* not reported, *RCT* randomized clinical trial, *UK* United Kingdom, *USA* United States of America

### Studies addressing exclusively patients undergoing foot and ankle surgery

Only four [17, 32, 34–36] of the 15 studies included patients undergoing foot and ankle surgery exclusively. Three [34–36] of these four studies were conducted in the UK and one [32] in Germany. Three [34–36] were observational studies, and one [32] had a quasi-experimental design. The included patients ranged from 56 [32] to 161 [35]. Regarding the primary outcome of each study, two evaluated the length of stay [34, 36], one evaluated the knowledge growth [32], and the other mainly assessed the readability and understanding of the preoperative information sheets [35]. The main characteristics, the evaluated education intervention, and the principal conclusions of the included studies assessing patients undergoing foot and ankle surgeries are shown in Table 2.

Overall, these four studies reported positive results: Speirs et al. [35] concluded that preoperative information sheets were well received by patients undergoing foot and ankle surgery, Thomas et al. [36] and Selvan et al. [34] demonstrated that preoperative education—in the form of foot school and a preoperative session respectively—reduced length of stay in the hospital, and Schafer et al. [32] concluded that patients’ knowledge and satisfaction improved with one formal session of preoperative education.

### Studies addressing patients undergoing any orthopedic surgery, including foot and ankle procedures

Eleven studies [25–31, 33, 37–39] included patients undergoing orthopedic surgery, including foot and ankle procedures. However, none of these studies presents separate data from patients undergoing specifically foot and ankle surgery, nor specify how many of their included participants underwent foot and ankle surgery.

All of these studies were published in English, three [27, 29, 30] were randomized clinical trials, five [26, 31, 37–39] had a quasi-experimental design, and the rest were observational studies [25, 28, 33]. The main characteristics of each study are shown in Table 3 and complemented with more detailed data in Additional file 4.

Regarding the primary outcome reported by each study, four evaluated [30, 37–39] the postoperative pain level, two [26, 27] specifically evaluated the use of opioids after surgery, and the others primarily assessed patient expectation [25], compliance [28], satisfaction [29], amount of information received [33], or empowerment [31].

## Discussion

Our study characterizes and resumes the existing literature after a broad and reproducible search in multiple databases and a careful screening and full-text selection process. This study is the first to map the available evidence of preoperative education for patients undergoing foot and ankle surgery. We found four [17, 32, 34–36] studies that specifically assessed different forms of preoperative education in patients undergoing foot and ankle surgery and 11 [25–31, 33, 37–39] studies that evaluated this in a broader population that included patients undergoing foot and ankle surgery, but that did not provide separate data of these patients.

Regarding the reported outcomes, as mentioned in the results section, the four studies including only foot and ankle surgery patients reported positive results with preoperative education. These results could vary with further studies because of the small sample sizes of the included studies. It is remarkable that the evaluated outcomes in all the included studies in our review varied widely (e.g., level of pain, length of stay, patient knowledge). This heterogeneity in the evaluated outcomes would make it very difficult to extrapolate, to compare, and—eventually—to combine the studies’ results. A way to homogenize the outcomes and to overcome the aforementioned difficulties could be to use patient-reported outcomes measures (PROM), which was already encouraged by the American Orthopedic Foot and Ankle Society (AOFAS) [40].

Furthermore, there was also a wide heterogeneity among each evaluated education intervention, going from foot school [36] to delivering a leaflet at the moment of informed consent [35]. As the interventions are heterogeneous and we did not find any study that compares different methods of education, the real impact of each of these educational methods remains to be determined. Some authors [17] have already suggested that preoperative education should consist of a live class to improve patient experience and reduce costs. This suggestion stands out from the diversity of educational interventions for all orthopedic surgeries, making it challenging to provide evidence-based recommendations.

Even though the existing evidence for preoperative education in foot and ankle surgery is scarce, evidence of preoperative education in other specialties of orthopedic surgery is slightly broader. It is outstanding that the four studies specifically addressing preoperative education for patients undergoing foot and ankle surgery were conducted in Europe. When comparing this with other orthopedics fields, already in 2014, a Cochrane systematic review published assessed preoperative education for hip or knee replacement [13], including 18 trials that were conducted mostly in Europe and North America (all randomized or quasi-randomized trials). This shows that evidence on preoperative education for other orthopedic surgeries is more substantial and of better quality than the included evidence regarding patients undergoing foot and ankle surgery in our study. However, for both patients undergoing foot and ankle surgeries or other orthopedic specialties, evidence regarding preoperative education comes from developed countries. In Cochrane’s systematic review mentioned above, despite the more extensive availability of data, the evidence for all the assessed outcomes—for both hip and knee replacement—was graded as low or very low quality of evidence, meaning that further studies are very likely to impact their estimated effects. Finally, the included studies in our review and the evidence for other orthopedic specialties make us question if preoperative education offers benefits over usual care. But, besides this reasonable doubt, it must be recognized that preoperative education might be a helpful adjunct with low risk of undesirable effects.

Further studies addressing preoperative education for patients undergoing foot and ankle surgery are needed to conclude about its effectiveness. We specifically suggest conducting prospective studies, either interventional or observational, on patients undergoing foot and ankle elective orthopedic surgery. First, these studies may compare a standardized educational method (e.g., foot school, nurse education session, video-recorded sessions) against the actual standard of care in a specific center (even if this is no formal educational). In places where a specific preoperative education tool is already in use, different formal education methods may be compared to address which tool is more effective. Regarding the evaluated outcomes in future studies, we encourage that future studies address PROMs as mentioned above; this may allow standardization of measurements and a combination of multiple studies results [40]. Authors should also measure patient satisfaction with the evaluated intervention, pain, use of analgesics (especially opioids), and length of hospital stay because all these outcomes are clinically relevant to decision-making. The outcomes should be measured at standardized time intervals, either preoperative when possible or postoperative. Taking into account these considerations, future studies may elucidate the real impact of preoperative education in this specific population.

A limitation of our study is that we did not assess the risk of bias in each included article. However, this was beyond the scope of our review, as we aimed to map, identify, and characterize all the existing evidence for preoperative education in patients undergoing foot and ankle surgery—which we properly accomplished.

## Conclusion

The available evidence on preoperative education for patients undergoing foot and ankle surgery is scarce and heterogeneous. However, this—very limited—evidence provides favorable outcomes regarding the length of hospital stay and patient knowledge and satisfaction. Further studies are needed to establish whether preoperative education positively impacts patients undergoing foot and ankle surgery and which methods are more effective for this purpose.

More studies addressing preoperative education in patients undergoing foot and ankle surgery are required to evidence-based recommend its use. With the (scarce) existent evidence, foot and ankle orthopedic surgeons should ensure that their patients receive preoperative education as it may decrease the length of stay in the hospital and increase the patient’s knowledge of the procedure.

### Supplementary Information

**Additional file 1.** Search strategies.

**Additional file 2. **Reports assessed for eligibility and reasons for exclusion.

**Additional file 3. **Cross citation (scholar google) and review of included studies references.

**Additional file 4. **Detailed data of studies addressing patients undergoing any orthopedic surgery, including foot and ankle procedures but not providing specific data of them.

## Data Availability

Not applicable.
