# Supplementary material for: Preoperative education in patients undergoing foot and ankle surgery: a scoping review
Source: Syst Rev. 2023 Nov 13;12:210. doi: 10.1186/s13643-023-02375-2 (PMC10644491; doi:10.1186/s13643-023-02375-2)
Supplement: Supplementary file 1 — Additional file 1. Search strategies. [file 13643_2023_2375_MOESM1_ESM.docx]

**Additional File 1. Search strategies**

**Medline.**

Ovid MEDLINE(R) and Epub Ahead of Print, In-Process, In-Data-Review & Other Non-Indexed Citations, Daily and Versions(R) <1946 to November 2021>

1 exp Preoperative Period/

2 exp Traumatology/

3 Traumatolog*.tw.

4 Surg*.tw.

5 1 or 2 or 3 or 4

6 exp Foot Joints/

7 Foot.ab,ti.

8 feet.ab,ti.

9 Ankle*.ti,ab.

10 tarsus.ab,ti.

11 metatarsus.ab,ti.

12 chopart.ab,ti.

13 forefoot.ab,ti.

14 Midfoot.ab,ti.

15 Leg.ab,ti.

16 calf.ab,ti.

17 orthopaedic*.ab,ti.

18 6 or 7 or 8 or 9 or 10 or 11 or 12 or 13 or 14 or 15 or 16 or 17

19 Patient Education.mp.

20 exp Patient Education as Topic/

21 exp Health Communication/

22 Health Communication.mp.

23 (patien$ adj2 (information or instruct$ or educat$ or Comunication$)).ti,ab.

24 19 or 20 or 21 or 22 or 23

25 5 and 18 and 24

**CENTRAL**

[Cochrane Database of Systematic Reviews](https://www.cochranelibrary.com/)

Issue 12 of 12, December 2021

ID Search Hits

#1 MeSH descriptor: [Traumatology] explode all trees

#2 (Traumatolog*):ti,ab,kw

#3 (Surg*):ti,ab,kw

#4 MeSH descriptor: [Preoperative Period] explode all trees

#5 #1 OR #2 OR #3 OR #4

#6 MeSH descriptor: [Foot Joints] explode all trees

#7 (Foot):ti,ab,kw

#8 (feet):ti,ab,kw

#9 (Ankle*):ti,ab,kw

#10 (tarsus):ti,ab,kw

#11 (metatarsus):ti,ab,kw

#12 (chopart):ti,ab,kw

#13 (forefoot):ti,ab,kw

#14 (Midfoot):ti,ab,kw

#15 (Leg):ti,ab,kw

#16 (calf):ti,ab,kw

#17 (orthopaedic*):ti,ab,kw

#18 #6 or #7 or #8 or #9 or #10 or #11 or #12 or #13 or #14 or #15 or #16 or #17

#19 (Patient Education):ti,ab,kw

#20 MeSH descriptor: [Patient Education as Topic] explode all trees 9214

#21 MeSH descriptor: [Health Communication] explode all trees 270

#22 (Health Communication):ti,ab,kw 9690

#23 ((patien$ NEAR/2 (information or instruct$ or educat$ or Comunication$))):ti,ab,kw 0

#24 #19 or #20 or #21 or #22 or #23 38533

#25 #5 and #18 and #24 317

**LILACS**

27.12.2021

(“Preoperative Period” OR Traumatolog* OR Surger*) AND (Joint OR Foot OR Feet OR metatarsus OR chopart OR leg OR calf OR ortopedic) AND (“patient information” OR “Patient Education”) (14)

**EMBASE**

**Elsevier.com**

#26. #5 AND #18 AND #24 AND [embase]/lim 926

#25. #5 AND #18 AND #24 1,142

#24. #19 OR #20 OR #21 OR #22 OR #23 228,227

#23. (patien$ NEAR/2 (information OR instruct$ OR 28,143

educat$ OR comunication$)):ti,ab

#22. 'health communication':ti,ab 3,031

#21. 'medical information'/exp 79,846

#20. 'patient education'/exp 118,742

#19. 'patient education':ti,ab 29,959

#18. #6 OR #7 OR #8 OR #9 OR #10 OR #11 OR #12 OR #13 458,905

OR #14 OR #15 OR #16 OR #17

#17. midfoot:ti,ab 3,291

#16. orthopaedic*:ti,ab 66,076

#15. calf:ti,ab 53,585

#14. leg:ti,ab 131,602

#13. forefoot:ti,ab 6,612

#12. chopart:ti,ab 363

#11. metatarsus:ti,ab 1,505

#10. tarsus:ti,ab 2,076

#9. ankle*:ti,ab 90,562

#8. feet:ti,ab 46,127

#7. foot:ti,ab 137,225

#6. 'foot joint'/exp 8,374

#5. #1 OR #2 OR #3 OR #4 3,107,170

#4. surg*:ti,ab 2,918,527

#3. traumatolog*:ti,ab 8,738

#2. 'traumatology'/exp 12,168

#1. 'preoperative period'/exp 366,717

**ERIC**

| S1 | TX (“Preoperative Period” OR Traumatolog* OR Surger*) AND (Joint OR Foot OR Feet OR metatarsus OR chopart OR leg OR calf OR ortopedic) AND (“patient information” OR “Patient Education”) (14) |
| --- | --- |

Trials:

**ICTRP:** preoperative AND education (2)

**Clinical trials.gov**:preoperative AND education (203)
