# Supplementary material for: Preoperative education in patients undergoing foot and ankle surgery: a scoping review
Source: Syst Rev. 2023 Nov 13;12:210. doi: 10.1186/s13643-023-02375-2 (PMC10644491; doi:10.1186/s13643-023-02375-2)
Supplement: Supplementary file 2 — Additional file 2. Reports assessed for eligibility and reasons for exclusion. [file 13643_2023_2375_MOESM2_ESM.docx]

## **Additional File 2. Reports assessed for eligibility and reasons for exclusion**

| ID study | Reason for exclusion |
| --- | --- |
| Badarqdeen 2010 [1] | Wrong intervention |
| Brown 2019 [2] | Wrong population |
| Dobberstein 1987 [3] | Wrong population |
| Heikkinen 2007 [4] | **INCLUDED** |
| Heikkinen 2008 [5] | Wrong population |
| Heikkinen 2009 [6] | Wrong population |
| Heikkinen 2012 a [7] | Wrong population |
| Heikkinen 2012 b [8] | Wrong population |
| Holman 2014 [9] | **INCLUDED** |
| Ilyas 2021 [10] | **INCLUDED** |
| Irwin 2021 [11] | Wrong intervention |
| Johansson 2005 [12] | Wrong design |
| Laude 2017 [13] | **INCLUDED** |
| Liu 2004 [14] | Wrong design |
| Mertz 2018 [15] | Wrong population |
| Morris 2014 [16] | **INCLUDED** |
| Noback 2021 [17] | Wrong intervention |
| Oliver 1999 [18] | Wrong intervention |
| Patteta 2021 [19] | Wrong intervention |
| Roussel 2019 [20] | Wrong population |
| Sagiv 2011 [21] | Wrong population |
| Sanchez 2016 [22] | Wrong population |
| Scott 2001 [23] | **INCLUDED** |
| Slover 2012 [24] | Wrong design |
| Speirs 2008 [25] | **INCLUDED** |
| Tomaszek 2019 [26] | Wrong population |
| Valkeapaa 2014 [27] | Wrong population |
| Van der Bauwhede 1998 [28] | Other reason: different aim and scope |
| Wong 2010 [29] | **INCLUDED** |
| Wongkietkachorn 2018 [30] | Wrong population |
| Zhou 2018 [31] | Wrong population |
| Allegaert Ongoing [32] | Other reason: authors did not described in the register which surgery the participants underwent. |
| Binay Ongoing [33] | Other reason: authors did not described in the register which surgery the participants underwent. |
| Bordeaux Ongoing [34] | Wrong population - Withdrawn |
| Bulich Ongoing [35] | Wrong population |
| Dziadzko Ongoing [36] | Other reason: Authors do not specified wether they are recruiting patients undergoing a foot and ankle surgery; "Patients admitted for an outpatient intervention resulting in moderate to severe pain: eg: arthroscopy of the knee or shoulder, cruciate ligaments, shoulder block, medial patellofemoral ligament plasty (not exhaustive)" |
| Gezginci Ongoing [37] | Wrong population |
| Greene Ongoing [38] | Wrong population |
| Matava Ongoing [39] | Other reason: Register did not precise which surgery were the children undergoing |
| McIsaac Ongoing [40] | Wrong population |
| Taylor Ongoing [41] | Wrong population |
| Wesson Ongoing [42] | Wrong population |

**References**

[1] Badarudeen S, Sabharwal S. Assessing readability of patient education materials: current role in orthopaedics. Clin Orthop Relat Res 2010;468:2572–80. https://doi.org/10.1007/s11999-010-1380-y.

[2] Brown OS, Toi TH, Barbosa PR, Pookarnjanamorakot P, Trompeter A. A patient-centred check sheet improves communication on the trauma ward round. Br J Hosp Med 2019;80:472–5. https://doi.org/10.12968/hmed.2019.80.8.472.

[3] Dobberstein K. Orthopaedic surgery: what patients need to know. Am J Nurs 1987;87:961.

[4] Heikkinen K, Leino-Kilpi H, Hiltunen A, Johansson K, Kaljonen A, Rankinen S, et al. Ambulatory orthopaedic surgery patients’ knowledge expectations and perceptions of received knowledge. J Adv Nurs 2007;60:270–8. https://doi.org/10.1111/j.1365-2648.2007.04408.x.

[5] Heikkinen K, Helena L-K, Taina N, Anne K, Sanna S. A comparison of two educational interventions for the cognitive empowerment of ambulatory orthopaedic surgery patients. Patient Educ Couns 2008;73:272–9. https://doi.org/10.1016/j.pec.2008.06.015.

[6] Heikkinen K, Salanterä S, Leino-Kilpi H. How do patients evaluate their education? - a comparison of two education methods. Stud Health Technol Inform 2009;146:850–1.

[7] Heikkinen K, Salanterä S, Leppänen T, Vahlberg T, Leino-Kilpi H. Ambulatory orthopaedic surgery patients’ emotions when using different patient education methods. J Perioper Pract 2012;22:226–31. https://doi.org/10.1177/175045891202200703.

[8] Heikkinen K, Leino-Kilpi H, Vahlberg T, Salanterä S. Ambulatory orthopaedic surgery patients’ symptoms with two different patient education methods. International Journal of Orthopaedic and Trauma Nursing 2012;16:13–20. https://doi.org/10.1016/j.ijotn.2011.04.004.

[9] Holman JE, Stoddard GJ, Horwitz DS, Higgins TF. The effect of preoperative counseling on duration of postoperative opiate use in orthopaedic trauma surgery: a surgeon-based comparative cohort study. J Orthop Trauma 2014;28:502–6. https://doi.org/10.1097/BOT.0000000000000085.

[10] Ilyas AM, Chapman T, Zmistowski B, Sandrowski K, Graham J, Hammoud S. The Effect of Preoperative Opioid Education on Opioid Consumption After Outpatient Orthopedic Surgery: A Prospective Randomized Trial. Orthopedics 2021;44:123–7. https://doi.org/10.3928/01477447-20210201-07.

[11] Irwin SC, Lennon DT, Stanley CP, Sheridan GA, Walsh JC. Ankle conFUSION: The quality and readability of information on the internet relating to ankle arthrodesis. Surgeon 2021;19:e507–11. https://doi.org/10.1016/j.surge.2020.12.001.

[12] Johansson K, Nuutila L, Virtanen H, Katajisto J, Salanterä S. Preoperative education for orthopaedic patients: systematic review. J Adv Nurs 2005;50:212–23. https://doi.org/10.1111/j.1365-2648.2005.03381.x.

[13] Laude K, Buchholz SW, Rodts M. Improving Patient Education at a Large Midwest Urban Orthopaedic Center. Orthop Nurs 2017;36:133–9. https://doi.org/10.1097/NOR.0000000000000327.

[14] Empowerment model of preoperative education for orthopaedic patients n.d. https://doi.org/10.1002/central/CN-01729699.

[15] Mertz K, Eppler S, Yao J, Amanatullah DF, Chou L, Wood KB, et al. Patient Perceptions Correlate Weakly With Observed Patient Involvement in Decision-making in Orthopaedic Surgery. Clin Orthop Relat Res 2018;476:1859–65. https://doi.org/10.1097/CORR.0000000000000365.

[16] Morris BJ, Richards JE, Archer KR, Lasater M, Rabalais D, Sethi MK, et al. Improving patient satisfaction in the orthopaedic trauma population. J Orthop Trauma 2014;28:e80–4. https://doi.org/10.1097/01.bot.0000435604.75873.ba.

[17] Noback PC, Trupia EP, Dziesinski LK, Sarpong NO, Trofa DP, Vosseller JT. Ankle Fractures: The Current State of Online Patient Information. Foot Ankle Spec 2021;14:324–33. https://doi.org/10.1177/1938640020916286.

[18] Oliver CW. Trauma and orthopaedic surgery on the Internet. J Bone Joint Surg Br 1999;81:3–6. https://doi.org/10.1302/0301-620x.81b1.9659.

[19] Patetta MJ, Pond KM, Tennant EM, Sood A, Gonzalez MH. Readability Level of English and Spanish Orthopaedic Patient Education Materials English and Spanish Patient Education. J Surg Orthop Adv 2021;30:96–100.

[20] Roussel S, Frenay M. Links Between Perceptions and Practices in Patient Education: A Systematic Review. Health Educ Behav 2019;46:1001–11. https://doi.org/10.1177/1090198119868273.

[21] Sagiv A. Planning treatment policy for school-aged children undergoing orthopaedic surgery with Ilizarov external fixation using the critical pathway. Int J Adolesc Med Health 2011;13:101–10. https://doi.org/10.1515/IJAMH.2001.13.2.101.

[22] Sanchez LM. Using the Power of 3 with Total Joint Surgery Patients. Walden University, 2016.

[23] Scott A. How Much Information is Too Much Information for Patients? Journal of Integrated Care Pathways 2001;5:119–25. https://doi.org/10.1177/147322970100500303.

[24] Slover J, Shue J, Koenig K. Shared decision-making in orthopaedic surgery. Clin Orthop Relat Res 2012;470:1046–53. https://doi.org/10.1007/s11999-011-2156-8.

[25] Speirs S, Rees S, Tagoe M. An audit of foot surgery information leaflets from the patients’ perspective. The Foot 2008;18:7–14. https://doi.org/10.1016/j.foot.2007.06.004.

[26] Tomaszek L, Cepuch G, Fenikowski D. Influence of preoperative information support on anxiety, pain and satisfaction with postoperative analgesia in children and adolescents after thoracic surgery: A randomized double blind study. Biomed Pap Med Fac Univ Palacky Olomouc Czech Repub 2019;163:172–8. https://doi.org/10.5507/bp.2018.060.

[27] Valkeapää K, Klemetti S, Cabrera E, Cano S, Charalambous A, Copanitsanou P, et al. Knowledge expectations of surgical orthopaedic patients: a European survey. Int J Nurs Pract 2014;20:597–607. https://doi.org/10.1111/ijn.12189.

[28] Van der Bauwhede J. The future of orthopaedics on the Internet. Acta Orthop Belg 1998;64:121–2.

[29] Wong EM-L, Chan SW-C, Chair S-Y. Effectiveness of an educational intervention on levels of pain, anxiety and self-efficacy for patients with musculoskeletal trauma. J Adv Nurs 2010;66:1120–31. https://doi.org/10.1111/j.1365-2648.2010.05273.x.

[30] Wongkietkachorn A, Wongkietkachorn N, Rhunsiri P. Preoperative Needs-Based Education to Reduce Anxiety, Increase Satisfaction, and Decrease Time Spent in Day Surgery: A Randomized Controlled Trial. World J Surg 2018;42:666–74. https://doi.org/10.1007/s00268-017-4207-0.

[31] Zhou L, Zhou L, Tian L, Zhu D, Chen Z, Zheng C, et al. Preoperative education with image illustrations enhances the effect of tetracaine mucilage in alleviating postoperative catheter-related bladder discomfort: a prospective, randomized, controlled study. BMC Anesthesiol 2018;18:204. https://doi.org/10.1186/s12871-018-0653-y.

[32] Kernel Networks Inc. The effectiveness of a preoperative, interactive game “SERES pain in children.” Case Medical Research 2019. https://doi.org/10.31525/ct1-nct03874442.

[33] Case Medical Research. Designing animated movie for preoperative period. Case Medical Research 2019. https://doi.org/10.31525/ct1-nct04176822.

[34] Impact of Videos on Patient Anxiety and Satisfaction n.d. https://clinicaltrials.gov/ct2/show/NCT03485937 (accessed May 17, 2023).

[35] PIRA vs Standard Preoperative Education n.d. https://clinicaltrials.gov/ct2/show/NCT04586569 (accessed May 17, 2023).

[36] Preoperative Education for Less Outpatient Pain After Surgery (PELOPS) - Full Text View - ClinicalTrials.Gov n.d. https://clinicaltrials.gov/ct2/show/NCT03754699 (accessed May 17, 2023).

[37] The Effectiveness of the Training Given With Audio and Picture Book Before the Surgery n.d. https://clinicaltrials.gov/ct2/show/NCT04960761 (accessed May 17, 2023).

[38] RCT: Impact of Preop Video on Patient Preparedness for Surgery n.d. https://clinicaltrials.gov/ct2/show/NCT02076360 (accessed May 17, 2023).

[39] Matava C, The Hospital for Sick Children. VR for Preoperative Anxiety in Children n.d. https://ichgcp.net/amp/clinical-trials-registry/NCT03201640 (accessed May 17, 2023).

[40] The prehabilitation study: Exercise Before Surgery to Improve Patient Function in People n.d. https://clinicaltrials.gov/ct2/show/NCT02934230 (accessed May 17, 2023).

[41] Patient Information Videos on Operations Trial - Full Text View - ClinicalTrials.Gov n.d. https://clinicaltrials.gov/ct2/show/NCT03689751 (accessed May 17, 2023).

[42] Kernel Networks Inc. Role of pre-operative counseling in the surgical patient. Case Medical Research 2019. https://doi.org/10.31525/ct1-nct03925961.
