# Supplementary material for: Preoperative education in patients undergoing foot and ankle surgery: a scoping review
Source: Syst Rev. 2023 Nov 13;12:210. doi: 10.1186/s13643-023-02375-2 (PMC10644491; doi:10.1186/s13643-023-02375-2)
Supplement: Supplementary file 3 — Additional file 3. Cross citation (scholar google) and review of included studies references. [file 13643_2023_2375_MOESM3_ESM.docx]

**Additional File 3. Cross citation (scholar google) and review of included studies references**

## Review of included studies references

| **Reference** |  |
| --- | --- |
| Speirs 2008 (1) | 32 references: 0 relevant studies |
| Heikkinen 2007 (2) | 34 references: 1 potentially relevant study   - **Pellino 1998** (3)**: included** |
| Holman 2014 (4) | 32 references: 0 relevant studies |
| Ilyas 2021 (5) | 18 references: 0 relevant studies |
| Johansson 2005 (6) | 41 references: 0 relevant studies |
| Laude 2017 (7) | 10 references: 0 relevant studies |
| Morris 2014 (8) | 12 references: 0 relevant studies |
| Scott 2001 (9) | 42 references: 0 relevant studies |
| Wong 2010a (10) | 80 references: 0 new relevant studies |
| Schafer 2017 (11) | 26 references:  -2 potentially relevant study:   - Johansson 2007 (12): excluded, wrong population - **Selvan 2013** (13)**: new included** |
| Majid 2015 (14) | 30 references: 0 new relevant studies |
| Wong 2014 (15) | 70 references: 0 new relevant studies |
| Pellino 1998 (3) | 32 references: 0 new relevant studies |
| Wong 2010b (16) | 8 references: 0 new relevant studies |
| Othin 2020 (17) | 38 references: 0 new relevant studies |
| Selvan 2013 (13) | 8 references: 0 new relevant studies |
| Thomas 2022 (18) | 18 references: 0 relevant studies |

## Cross citation (Scholar google)

| **Reference** |  |
| --- | --- |
| Speirs 2008 (1) | 14-06-2022  Cited by 4: 0 relevant study |
| Heikkinen 2007 (2) | 18-06-2022  Cited by 88: 3 potentially relevant studies. 1 new included study   - Heinneken 2008 (19): already excluded, wrong population - Heinneken 2011 (20): already excluded, wrong population - **Schäfer 2017** (11)**: new included** |
| Holman 2014 (4) | 18-06-2022  Cited by 72: 1 potentially relevant study   - Ilyas 2021 (5): already included |
| Ilyas 2021 (5) | 18-06-2022  Cited by 6: 0 relevant studies |
| Johansson 2005 (6) | 18-06-2022  Cited by 323: 7 potentially relevant studies. 1 new included study   - Wong 2010a (10): already Included - Majid 2015 (14): Excluded, wrong study design (systematic review protocol) - **Wong 2010b** (16)**: new included** - Heinneken 2012 (21): already excluded, wrong population - Heinneken 2011 (20)): already excluded, wrong population - **Wong 2014** (15)**: new included** - Dziadzko 2022 (22): excluded, wrong population |
| Laude 2017 (7) | 18-06-2022  Cited by 2: 0 relevant studies |
| Morris 2014 (8) | 18-06-2022  Cited by 23: 0 relevant studies |
| Scott 2001 (9) | 18-06-2022  Cited by 7: 0 relevant studies |
| Wong 2010a (10) | 18-06-2022  Cited by 151: 0 new potentially relevant studies |
| Schafer 2017 (11) | 18-06-2022  Cited by 2: 0 relevant studies |
| Wong 2014 (15) | 18-06-2022  Cited by 18: 0 relevant studies |
| Pellino 1998 (3) | 19-06-2022:  Cited by 158: 3 potentially relevant studies. 1 new included study.   - Dekkers 2018 (23): excluded, wrong population. - Lewis 2002 (24): excluded, wrong population - **Othin 2020** (17)**: new included** |
| Wong 2010b (16) | 19-06-2022:  Cited by 30: 0 relevant studies |
| Othin 2020 (17) | 19-06-2022:  Cited by 0 |
| Selvan 2013 (13) | 17-07-2022:  Cited by 9: 1 potentially relevant study. 1 new included study.   - **Thomas 2022** (18)**: new included** |
| Thomas 2022 (18) | 17-07-2022  Cited by 0 |

1. Speirs S, Rees S, Tagoe M. An audit of foot surgery information leaflets from the patients’ perspective [Internet]. Vol. 18, The Foot. 2008. p. 7–14. Available from: http://dx.doi.org/10.1016/j.foot.2007.06.004

2. Heikkinen K, Leino-Kilpi H, Hiltunen A, Johansson K, Kaljonen A, Rankinen S, et al. Ambulatory orthopaedic surgery patients’ knowledge expectations and perceptions of received knowledge. J Adv Nurs [Internet]. 2007 Nov;60(3):270–8. Available from: http://dx.doi.org/10.1111/j.1365-2648.2007.04408.x

3. Pellino T, Tluczek A, Collins M, Trimborn S, Norwick H, Engelke ZK, et al. Increasing self-efficacy through empowerment. Orthop Nurs [Internet]. 1998 Jul;17(4):48???59. Available from: http://journals.lww.com/00006416-199807000-00009

4. Holman JE, Stoddard GJ, Horwitz DS, Higgins TF. The effect of preoperative counseling on duration of postoperative opiate use in orthopaedic trauma surgery: a surgeon-based comparative cohort study. J Orthop Trauma [Internet]. 2014;28(9):502–6. Available from: http://dx.doi.org/10.1097/BOT.0000000000000085

5. Ilyas AM, Chapman T, Zmistowski B, Sandrowski K, Graham J, Hammoud S. The Effect of Preoperative Opioid Education on Opioid Consumption After Outpatient Orthopedic Surgery: A Prospective Randomized Trial. Orthopedics [Internet]. 2021;44(2):123–7. Available from: http://dx.doi.org/10.3928/01477447-20210201-07

6. Johansson K, Nuutila L, Virtanen H, Katajisto J, Salanterä S. Preoperative education for orthopaedic patients: systematic review. J Adv Nurs [Internet]. 2005;50(2):212–23. Available from: http://dx.doi.org/10.1111/j.1365-2648.2005.03381.x

7. Laude K, Buchholz SW, Rodts M. Improving Patient Education at a Large Midwest Urban Orthopaedic Center. Orthop Nurs [Internet]. 2017;36(2):133–9. Available from: http://dx.doi.org/10.1097/NOR.0000000000000327

8. Morris BJ, Richards JE, Archer KR, Lasater M, Rabalais D, Sethi MK, et al. Improving patient satisfaction in the orthopaedic trauma population. J Orthop Trauma [Internet]. 2014;28(4):e80–4. Available from: http://dx.doi.org/10.1097/01.bot.0000435604.75873.ba

9. Scott A. How Much Information is Too Much Information for Patients? Journal of integrated Care Pathways [Internet]. 2001 Dec 1;5(3):119–25. Available from: https://doi.org/10.1177/147322970100500303

10. Wong EML, Chan SWC, Chair SY. Effectiveness of an educational intervention on levels of pain, anxiety and self-efficacy for patients with musculoskeletal trauma. J Adv Nurs [Internet]. 2010;66(5):1120–31. Available from: http://dx.doi.org/10.1111/j.1365-2648.2010.05273.x

11. Schäfer A, Jettkowski K, Kretschmann J, Wurg M, Stukenborg-Colsmann C, Plaaß C. Development and evaluation of interdisciplinary preoperative patient education in foot and ankle surgery: immediate effects on knowledge, satisfaction and anxiety / Entwicklung und erste Evaluation eines präoperativen interdisziplinären Schulungskonzeptes für Patienten/-innen der Fußchirurgie: unmittelbare Effekte auf Wissenszuwachs, Zufriedenheit und Angst [Internet]. Vol. 4, International Journal of Health Professions. 2017. p. 25–32. Available from: http://dx.doi.org/10.1515/ijhp-2017-0005

12. Johansson K, Salanterä S, Katajisto J. Empowering orthopaedic patients through preadmission education: results from a clinical study. Patient Educ Couns [Internet]. 2007 Apr;66(1):84–91. Available from: http://dx.doi.org/10.1016/j.pec.2006.10.011

13. Selvan D, Molloy A, Abdelmalek A, Mulvey I, Alnwick R. The effect of preoperative foot and ankle physiotherapy group on reducing inpatient stay and improving patient care. Foot Ankle Surg [Internet]. 2013 Jun;19(2):118–20. Available from: http://dx.doi.org/10.1016/j.fas.2012.12.004

14. Majid N, Lee S, Plummer V. The effectiveness of orthopedic patient education in improving patient outcomes: a systematic review protocol. JBI Database System Rev Implement Rep [Internet]. 2015 Jan;13(1):122–33. Available from: http://dx.doi.org/10.11124/jbisrir-2015-1950

15. Wong EML, Chair SY, Leung DYP, Chan SWC. Can a brief educational intervention improve sleep and anxiety outcomes for emergency orthopaedic surgical patients? [Internet]. Contemporary Nurse. 2014. p. 4292–321. Available from: http://dx.doi.org/10.5172/conu.2013.4292

16. Wong EML, Chan SWC, Chair SY. The effect of educational intervention on pain beliefs and postoperative pain relief among Chinese patients with fractured limbs. J Clin Nurs [Internet]. 2010 Sep;19(17-18):2652–5. Available from: http://dx.doi.org/10.1111/j.1365-2702.2010.03260.x

17. Othin M, Sendagire C, Mukisa J, Lubulwa C, Mulepo P, Wabule A, et al. Effect of preoperative information about pain on postoperative pain experience and patient satisfaction following orthopaedic surgery: A randomised controlled trial [Internet]. Research Square. Research Square; 2020. Available from: https://www.researchsquare.com/article/rs-130942/v1

18. Thomas T, Khan S, Saldanha K, Ballester JS, Stott R, Morgan S. Foot school: Preoperative education before day case elective foot and ankle surgery reduces the length of stay after surgery. Foot [Internet]. 2022 Mar;50:101893. Available from: http://dx.doi.org/10.1016/j.foot.2021.101893

19. Heikkinen K, Helena LK, Taina N, Anne K, Sanna S. A comparison of two educational interventions for the cognitive empowerment of ambulatory orthopaedic surgery patients. Patient Educ Couns [Internet]. 2008 Nov;73(2):272–9. Available from: http://dx.doi.org/10.1016/j.pec.2008.06.015

20. Heikkinen K, Salanterä S, Suomi R, Lindblom A, Leino-Kilpi H. Ambulatory orthopaedic surgery patient education and cost of care. Orthop Nurs [Internet]. 2011 Jan;30(1):20–8. Available from: http://dx.doi.org/10.1097/NOR.0b013e318205747f

21. Heikkinen K, Salanterä S, Leppänen T, Vahlberg T, Leino-Kilpi H. Ambulatory orthopaedic surgery patients’ emotions when using different patient education methods. J Perioper Pract [Internet]. 2012;22(7):226–31. Available from: http://dx.doi.org/10.1177/175045891202200703

22. Dziadzko M, Bouteleux A, Minjard R, Harich J, Joubert F, Pradat P, et al. Preoperative Education for Less Outpatient Pain after Surgery (PELOPS trial) in orthopedic patients-study protocol for a randomized controlled trial. Trials [Internet]. 2022 May 21;23(1):422. Available from: http://dx.doi.org/10.1186/s13063-022-06387-6

23. Dekkers T, Melles M, Groeneveld BS, de Ridder H. Web-Based Patient Education in Orthopedics: Systematic Review. J Med Internet Res [Internet]. 2018 Apr 23;20(4):e143. Available from: http://dx.doi.org/10.2196/jmir.9013

24. Lewis C, Gunta K, Wong D. Patient Knowledge, Behavior, and Satisfaction With the Use of a Preoperative DVD. Orthop Nurs [Internet]. 2002 Nov [cited 2022 Jun 19];21(6):41. Available from: https://journals.lww.com/orthopaedicnursing/Fulltext/2002/11000/Patient_Knowledge,_Behavior,_and_Satisfaction_With.9.aspx?casa_token=YoJe1y7ABO0AAAAA:5PJoi0jLoRa5re3I7x10cWks_xdk_Lgd-uuVqQ4mX1N9j5ahvIufCmBadCZg8dQFnhjrJ_WKCtDkIZU_hCwWzTh7ME0
